# Spatial distribution of pathogenic fungal isolates from clinical samples in Uganda: Diagnostic gaps and trends, January 2020 - May 2024

**DOI:** 10.1371/journal.pone.0327968

**Published:** 2025-07-17

**Authors:** Priscilla Atim, Samuel Gidudu, Bernard Ssentalo Bagaya, Andrew Kambugu, Grace Najjuka, Atuhaire D. Winfred, Benedict Kanamwanji, Esther Nabende, Adella Atukunda, Jonathan Kabazzi, Sylvia Joyoo, Hildah Tendo Nansikombi, Alex Riolexus Ario

**Affiliations:** 1 Uganda Public Health Fellowship Program, National Institute of Public Health, Kampala Uganda; 2 Infectious Diseases Institute, Makerere University, Kampala, Uganda; 3 Department of Immunology and Molecular Biology, Makerere University, Kampala, Uganda; 4 National Microbiology Reference Laboratory, Uganda National Health Laboratory and Diagnostic Services, Kampala, Uganda; Gulu University, UGANDA

## Abstract

**Background:**

Pathogenic fungi cause approximately 13 million infections and 1.5 million deaths worldwide each year, yet surveillance and diagnosis remain inadequate in resource-limited settings. In Uganda, fungal infections affect approximately 4,099,357 per 45 million people annually, resulting in severe invasive diseases if untreated. This study describes laboratory-confirmed pathogenic fungal isolates from clinical samples in Uganda from January 2020 to May 2024, and highlights gaps in diagnostic capacity.

**Methods:**

We abstracted data from the National Microbiology Reference Laboratory database, disaggregated pathogenic fungal isolates by the sex and age group of the patients, sample type, and isolated species, district, and year of isolation. Pathogenic fungal isolates were confirmed by culture and biochemical tests. Using Epi Info 7 software, we analyzed frequencies.

**Results:**

Among 8,136 clinical samples tested, fungal pathogens were isolated from 744 (9%) samples. Of these, the majority were obtained from female (92%), persons aged 16–35 years (68%). Most fungal pathogens (93.7%) were isolated from superficial clinical samples, while 6.3% from deep samples. High-vaginal swabs accounted for 71% of the clinical samples, with most cases from Kampala (32%) and Mbarara (26%) districts. The pathogenic fungal species identified included *C. albicans* (65.4%), non-*albicans Candida* spp. (30.6%) and *C. neoformans* (3.9%). We observed a sharp decline of identified pathogenic fungi from 299 (40%) in 2020–39 (5%) in 2024, reflecting diagnostic disruptions during the COVID-19 pandemic.

**Conclusion:**

*Candida* spp. were the most commonly isolated pathogenic fungi, mainly among females and individuals aged 16–35 years from Kampala and Mbarara districts. There is need for targeted interventions against candidiasis in these groups and locations. This study also highlights the gaps in fungal diagnostic capacity in Uganda, as the national database was limited to *Candida* and *Cryptococcus*, emphasizing the need for improved diagnostic infrastructure, capacity-building and surveillance to enhance detection of pathogenic fungi.

## Introduction

Pathogenic fungi cause a spectrum of fungal infections, ranging from superficial to life-threatening invasive diseases [1]. Globally, they are responsible for a staggering 13 million fungal infections and 1.5 million deaths each year, especially among immunocompromised patients [2]. In 2024, the annual burden of fungal infections in Uganda was estimated to be approximately 4,099,357 cases [3]. These infections are common opportunistic infections associated with chronic diseases such as HIV, tuberculosis, diabetes mellitus, chronic lung diseases, cancer and asthma [4]. They also frequently affect severely ill patients in intensive care units, patients undergoing invasive medical procedures, and those taking immunosuppressants and broad-spectrum antibiotics [4]. Although fungal infections are commonly reported in immunocompromised patients [5], emerging evidence indicates an increasing number of cases in immunocompetent individuals [6–8]. However, the actual burden of fungal infections in Uganda remains unknown due to lack of comprehensive diagnostic capacity and surveillance.

In response to the growing threat of fungal pathogens, the World Health Organization (WHO) has increased its efforts to improve the surveillance of pathogenic fungi. As a result, WHO published its first fungal pathogen priority list in 2022 [9]. On the critical list are *C. albicans, C. auris, C. neoformans* and *A. fumigatus*; while other high-priority pathogens include *C. glabrata, Histoplasma* spp.*, Mucorales, Fusarium* spp*., C. tropicalis,* and *C. parapsilosis* [9]. Among these, the *Candida* spp.*, C. neoformans* and *A. fumigatus* are the leading causes of fungal-related morbidity and mortality in humans [10]. In Uganda, *Candida* spp. are often isolated from high vaginal swabs, while and *C. neoformans* is commonly isolated from cerebrospinal fluid samples, in HIV patients [3].

Despite initiatives such as AIDS control programs that have improved outcomes for HIV/AIDS patients with opportunistic infections [11], inadequate fungal diagnostics remain a critical barrier to effective disease management. In Regional Referral Hospital laboratories (RRHs), fungal diagnosis relies on direct microscopy, culture and Cryptococcal Antigen Lateral flow assays (CrAg). At the National Microbiology Reference Laboratory (NMRL), fungal pathogens are confirmed by culture and biochemical tests. However, the laboratories do not perform antifungal susceptibility testing due to lack of infrastructure. These constraints not only contribute to underreporting and misdiagnosis [12] but also inadequate public health interventions [13,14].

This study describes laboratory-confirmed pathogenic fungi isolated from clinical samples in Uganda from January 2020 to May 2024, highlighting the need for improved diagnostic infrastructure and surveillance.

## Methods

### Study design and setting

We conducted a descriptive analysis of laboratory-confirmed pathogenic fungal isolates from January 2020 to May 2024. Data were obtained from the National Microbiology Reference Laboratory (NMRL) database. The NMRL, a government laboratory accredited by the College of American Pathologists (CAP), conducts routine sample testing requested by clinicians in Kampala and Wakiso districts which serve as central healthcare hubs. Additionally, NMRL performs quality checks by retesting isolates received from all regional referral hospitals (RRHs) in Uganda, ensuring the quality and completeness of the national fungal pathogen dataset.

### Procedure for fungal detection in Uganda

Samples are referred to the NMRL using a national microbiology request form. In regional referral hospitals, fungi are diagnosed in the laboratory using direct microscopy, culture and CrAg tests, which are cost-effective methods commonly available in RRHs. Samples are then referred to NMRL for confirmation and further identification. If the RRHs do not have preliminary testing capacity, the samples are still directly referred to the NMRL. Once received, patient data are transferred to the NMRL information system, which automatically assigns a unique laboratory identification. At NMRL, fungi are identified using a combination of culture on Sabouraud Dextrose Agar (SDA), germ tube testing to differentiate *C. albicans*, India ink for *C. neoformans*, biochemical urea testing for urease-producing fungi.

Clinical sample types were also categorized into superficial and deep samples. Superficial samples were obtained from non-sterile body sites, including cutaneous samples, mucosal samples, catheter swabs and urine samples. Deep samples were collected from sterile internal body sites, such as CSF, blood, pleural fluid.

### Data abstraction, sources and analysis

We abstracted data on pathogenic fungal isolates from January 2020 to May 2024 from the NMRL database. The data included the sex and age group of the patients, sample type, isolated fungal species, district, and year of isolation. These data were then entered into Epi Info 7 software for analysis. We computed the frequency and proportion of confirmed pathogenic fungal isolates by the sex and age group of the patients, isolated species, sample type, district and year of isolation.

All data were accessed for research purposes between 01/05/2024 and 30/05/2024. No Personal Identifiable Information (PID) was used in this study, as all PIDs were blinded and replaced with laboratory-generated code numbers to ensure anonymity. Data were securely stored within NMRL, with restricted access requiring authorization.

### Ethical considerations and consent to participate

Our study utilized routinely generated secondary aggregated data with no personal identifiers in health facility outpatient and in-patient monthly reports, obtained from the NMRL database. The Uganda Public Health Fellowship Program operates under the Ministry of Health (MoH) and is part of the National Rapid Response Team, and has been granted permission to access and analyze surveillance data in the DHIS-2 and other data such as survey and field investigation data to inform decision making in the control and prevention of outbreaks and public health programming. Additionally, the MOH has also granted the program permission to disseminate the information through scientific publications. This analysis was authorized by MoH and classified as non-research, and as per our memorandum of understanding, it was exempt from full Institutional Review Board review, primarily aimed at public health practice. We stored the abstracted data set in a password-protected computer and only shared it with the investigation team. This study adhered to the principles outlined in the declaration of Helsinki and was conducted in accordance with national guidelines. All procedures involving human data were performed in compliance with these ethical standards.

## Results

### Description of confirmed pathogenic fungal cases

Among the 8,136 clinical samples tested, pathogenic fungi were confirmed in 744 (9%) samples from January 2020–May 2024. Among the 744 confirmed fungal isolates, most were from female (92%), aged 16–35 years (68%). *C. albicans* was the most common (65.4%) Table 1.

**Table 1:** Confirmed pathogenic fungal isolates in Uganda, January 2020-May 2024.

Variable	Frequency (n = 744)	Proportion (%)
**Sex**		
Female	681	92
Male	63	8
**Age group in years**		
0-15	51	6.8
16-35	502	68
36-60	161	21.6
≥ 60	30	4
**Pathogenic yeast species**		
*Candida albicans*	487	65.4
Non-*albicans Candida* spp.	228	30.6
*Cryptococcus neoformans*	29	3.9

### Pathogenic fungal species isolated by sample type

*Candida* and *Cryptococcus* were the only fungal genera isolated. The majority of the laboratory-confirmed pathogenic fungal isolates (526/744, 71%) were detected in high vaginal swab (HVS) samples (Table 2). Of all analyzed clinical samples, 93.7% were classified as superficial, while 6.3% were classified as deep. Cutaneous samples were not present in the dataset. *C. albicans* was predominantly detected in superficial samples, whereas *C. neoformans* was detected only in deep samples (CSF). Isolates from HVS samples included *C. albicans* (378/487, 78%) and non-*albicans Candida* spp. (148/228, 65%). The laboratory did not have the capacity to identify specific non*-albicans Candida* spp. All CSF samples that tested positive for fungal infections (29 (100%) were infected with *C. neoformans* (Table 2).

**Table 2:** Pathogenic fungal isolates by sample type in Uganda from January 2020 to May 2024.

Sample type	*C. albicans N = 487 (%)*	Non*-albicans* *N = 228 (%)*	*C. neoformans* *N = 29 (%)*	Total *N = 744 (%)*
**Superficial Samples**				697 (93.7)
Wound swab	4 (1)	3 (1)	0 (0)	7 (0.9)
Pus swab	5 (1)	2 (1)	0 (0)	7 (0.9)
Ear swab	6 (1)	3 (1)	0 (0)	9 (1.2)
Endocervical swab	1 (0)	0 (0)	0 (0)	1 (0.1)
Gastric Aspirate	1 (0)	0 (0)	0 (0)	1 (0.1)
Throat swab	1 (0)	1 (0)	0 (0)	2 (0.3)
Stool	2 (0)	0 (0)	0 (0)	2 (0.3)
Urethral swab	3 (1)	1 (0)	0 (0)	4 (0.5)
Cervical Swab	5 (1)	2 (1)	0 (0)	7 (0.9)
Sputum	14 (3)	10 (4)	0 (0)	24 (3.2)
Urine	58 (12)	49 (21)	0 (0)	107 (14.4)
High Vaginal Swab	378 (78)	148 (65)	0 (0)	526 (70.7)
**Deep Samples**				47(6.3)
Pleural Fluid	0 (0)	1 (0)	0 (0)	1 (0.1)
Blood	5 (1)	3 (1)	0 (0)	8 (1.1)
Catheter Urine	4 (1)	5 (2)	0 (0)	9 (1.2)
CSF	0 (0)	0 (0)	29 (100)	29 (3.9)

Percentages for *C. albicans, non-albicans Candida* spp. and *C. neoformans* are calculated within their respective subgroups (N = 487 for *C. albicans*, N = 228 for *non-albicans Candida* and N = 29 for *C. neoformans*). Percentages in the ‘Total’ column reflect overall distribution across all samples (N = 744).

**Fig 1:**
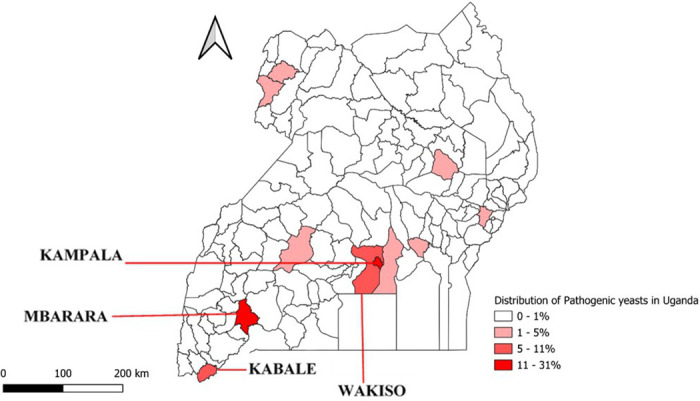
Proportion of confirmed pathogenetic fungal isolates by district in Uganda from January 2020–May 2024. (Map drawn using QGIS 3.32.2, link to base layer shape file. https://www.naturalearthdata.com/downloads/).

The percentage of confirmed pathogenic fungal isolates by district ranged from 0–32%. The majority of confirmed pathogenic fungal isolates were from Kampala (32%) and Mbarara (26%) districts (Fig 1).

**Fig 2:**
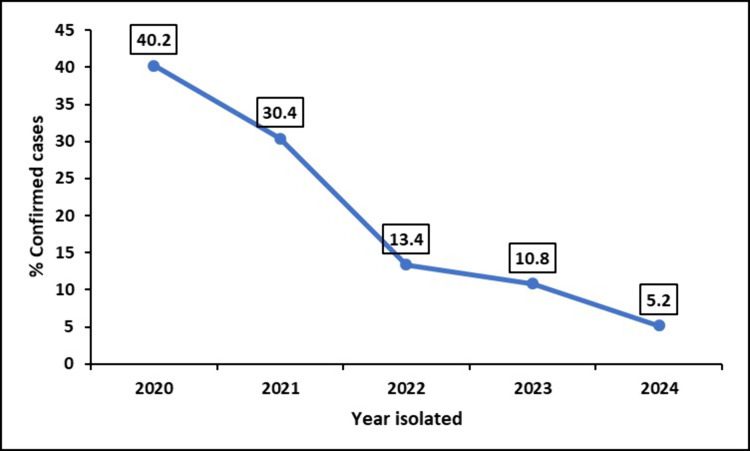
Proportion of confirmed pathogenic fungal isolates over time in Uganda from 2020 to 2024.

We observed a gradual decline in the number of confirmed pathogenic fungal isolates from 40% in 2020 to 5% in 2024 (Fig 2).

## Discussion

This study identified a sex, age and geographical disparity in the distribution of pathogenic fungal isolates in Uganda, with most cases detected among females and youths. The majority of pathogenic fungal isolates in our dataset were among persons from Kampala and Mbarara districts.

Our findings confirm that *Candida* spp., remain the most predominant fungal pathogens in Uganda [15], accounting for 66% of all isolates. While these results are consistent with global trends, [16,17], they highlight the need for improved diagnostic capacity to identify and manage non-*albicans Candida* spp., which are emerging as significant pathogens with varied antifungal resistance patterns [17–19]. Although the laboratory was able to identify *C. albicans*, it lacked the capacity to identify other *Candida* spp. or perform antifungal testing. Clinical fungal diagnosis in Ugandan laboratories is largely limited to the genus level of *Candida* and *Cryptococcus*, with no diagnostic capacity for other clinically significant fungi. This gap in diagnosis impedes effective clinical management and antifungal stewardship [3].

*C. neoformans* was identified in all the CSF samples that tested positive. Previous studies in Uganda have shown a high prevalence of cryptococcosis as an opportunistic infection in HIV patients with a rate of 60% [20]. Our results were consistent with these estimates. While many HIV/AIDS patients are diagnosed and treated through AIDS control programs that have been established in the country [11], these findings emphasize the need to expand diagnostic capacity and provide additional support to the National Microbiology Reference Laboratory [11]. This would ensure consistent and accessible diagnosis of cryptococcal meningitis across the healthcare system.

Our results showed that the majority of pathogenic fungal isolates were from females, consistent with the literature suggesting higher susceptibility among females to candida yeast infections [21], particularly during childbearing age [22]. Hormonal changes during pregnancy or from obesity, as well as the use of broad-spectrum antibiotics and high-dose estrogen birth control pills, contribute to this susceptibility [23]. While candida infections are common in sexually active women, data on sexually transmitted candida infections remain insufficient [21]. The dataset that we analyzed did not provide details about the sexual activity of this population. However, persons aged 16–35 years, who account for the highest proportion of infections, are typically sexually active. This disparity underscores the need for targeted education and preventive measures against candidiasis for women, especially those in high-risk groups.

Fungal pathogens were isolated from both localized superficial clinical samples and invasive deep samples. *Candida* spp. were isolated from all superficial clinical samples, which represent the most common sample type associated with candidiasis in both healthy and immunocompromised individuals [24]. The high proportion of isolates from high vaginal swabs correlates with the high number of female patient cases in the study dataset. Urine samples accounted for 14% of the pathogenic fungal isolates, suggesting vaginal and urinary tract yeast colonization. While yeast urinary tract infections are rare in healthy individuals, they are commonly detected in hospitalized patients and those with underlying medical conditions [25]. Candidemia caused by both *albicans* and non*-albicans Candida* was reported in 1% of the samples. The mortality rate from candidemia remains high, ranging from approximately 46% to 70% [26,27]. In addition, candidemia is associated with longer hospital stays and increased physical complications, which can be averted with early diagnosis and treatment [28]. While the overall burden of candidemia remains unknown in Uganda, our findings indicate that *C. albicans* is the most frequently isolated fungal species from bloodstream infections [29]. Other sample types, including CSF, sputum, and stool, and various swabs (ear, cervix, pus, wound, and urethra) each contributed less than 5% of the total isolates, highlighting the different clinical sources from which fungal pathogens were isolated. Most of these were among persons from Kampala and Mbarara districts, suggesting not only a regional hotspot but also the presence of access to healthcare facilities in these urban centers for fungal infections that warrants further investigation. In addition, Kampala and Mbarara have the most healthcare facilities in the country, thus improving access to healthcare [30]. This, together with more health-seeking behavior, could have contributed to the high proportion of samples from Kampala and Mbarara districts.

The number of confirmed pathogenic fungal isolates decreased from 40% in 2020 to 5% in 2024. While part of this decline can be attributed to the limited data collection period in 2024 only up to May, the COVID-19 pandemic played a significant role in disrupting routine diagnostics and laboratory services. During this time, resources were reallocated to address SARS-CoV-2 testing, leading to reduced surveillance for other infections, increased misdiagnosis, and reliance on over-the-counter medications [14,31,32]. This disruption highlights the fragility of diagnostic systems in low- and middle-income countries, and emphasizes the need to build resilient national healthcare infrastructure capable of maintaining routine surveillance during public health emergencies [33].

## Study limitations

Our findings were limited to fungi genera (*Candida* and *Cryptococcus*) due to the diagnostic capacity of the National Microbiology Reference Laboratory. The laboratory lacked the resources and infrastructure to specifically identify non-*albicans Candida* spp. and other fungal pathogens, such as *Aspergillus* spp*., Histoplasma* spp., which may also contribute to morbidity. Cutaneous samples were not present in the dataset, showing a diagnostic focus on systemic infections rather than cutaneous and dermatological fungal isolates. This absence may contribute to a lower estimate of the overall fungal infection burden. In addition, the laboratory lacks capacity to perform antifungal susceptibility testing, creating a gap in understanding the true burden of these fungal infections [34]. This highlights the need to expand fungal diagnostic capacity in Uganda to improve the detection and comprehensive management of clinical fungal pathogens.

## Conclusions

This study highlights a high proportion of *Candida* spp. isolates among females, persons aged 16–35 years, and those in Kampala and Mbarara districts. There is need to improve diagnostic capacity and establish surveillance systems for pathogenic fungal isolates in Uganda.

We recommend establishing a dedicated and equipped national mycology program to enhance diagnostic capacity and surveillance. The Ministry of Health Uganda would benefit from prioritizing resource allocation, staff training, and the provision of necessary equipment to build this capacity. This will enable comprehensive data collection to assess the true burden of fungal infections in the country and facilitate effective public health interventions. Additionally, it will align with global efforts to combat the rising threat of fungal pathogens.

## Supporting information

S1 Dataset Dataset used for analysis of pathogenic fungal isolates from clinical samples in Uganda (January 2020 - May 2024). (XLS)
